# Autobiographical Memory Disturbances in Depression: A Novel Therapeutic Target?

**DOI:** 10.1155/2015/759139

**Published:** 2015-08-25

**Authors:** Cristiano A. Köhler, André F. Carvalho, Gilberto S. Alves, Roger S. McIntyre, Thomas N. Hyphantis, Martín Cammarota

**Affiliations:** ^1^Memory Research Laboratory, Brain Institute, Federal University of Rio Grande do Norte (UFRN), 59056-450 Natal, RN, Brazil; ^2^Translational Psychiatry Research Group and Department of Clinical Medicine, Federal University of Ceara, 60430-140 Fortaleza, CE, Brazil; ^3^Departments of Psychiatry and Pharmacology, University of Toronto, Toronto, ON, Canada M5T 2S8; ^4^Mood Disorders Psychopharmacology Unit, University Health Network, University of Toronto, Toronto, ON, Canada M5T 2S8; ^5^Department of Psychiatry, University of Ioannina, 45110 Ioannina, Greece

## Abstract

Major depressive disorder (MDD) is characterized by a dysfunctional processing of autobiographical memories. We review the following core domains of deficit: systematic biases favoring materials of negative emotional valence; diminished access and response to positive memories; a recollection of overgeneral memories in detriment of specific autobiographical memories; and the role of ruminative processes and avoidance when dealing with autobiographical memories. Furthermore, we review evidence from functional neuroimaging studies of neural circuits activated by the recollection of autobiographical memories in both healthy and depressive individuals. Disruptions in autobiographical memories predispose and portend onset and maintenance of depression. Thus, we discuss emerging therapeutics that target memory difficulties in those with depression. We review strategies for this clinical domain, including memory specificity training, method-of-loci, memory rescripting, and real-time fMRI neurofeedback training of amygdala activity in depression. We propose that the manipulation of the reconsolidation of autobiographical memories in depression might represent a novel yet largely unexplored, domain-specific, therapeutic opportunity for depression treatment.

## 1. Introduction

Depression is a chronic and devastating mental disorder with an estimated lifetime prevalence of 11.1–14.6% worldwide [1]. This disorder significantly impacts workforce performance [2] and is associated with significant risks for all-cause and suicide mortality [3]. Cognitive models for depression provide a framework for comprehension of the psychological mechanisms associated with its onset and recurrence. One of the most influential of these models is the negative cognitive triad proposed by Beck [4], which suggests that depression results from activation of dysfunctional negatively biased schemas about the self, the world, and the future. Schemas in turn drive negatively biased cognitive processes, which in a vicious cycle consolidate the schemas and provide the cognitive roots for perpetuating the disorder [4]. Beck's model conceptualizes biases and distortions in cognitive processes as rational and adaptive mechanisms which become maladaptive and disturbed in chronic mental disorders [4]. This theoretical paradigm supports cognitive behavioral therapy (CBT), a psychotherapy that seeks identifying and modifying the biases in cognitive processes and ultimately transform maladaptive cognitive schemas to more functional ones [5]. Compelling evidences indicate that CBT is effective for depression treatment [6].

Consolidated memories were once thought to be persistent and resistant to disruption [7]. However, accumulating evidence has challenged this hypothesis by showing that recollection returns consolidated memories to a labile state [8–10] and that in order to persist these reactivated memories must undergo a protein synthesis-dependent process referred to as reconsolidation [10]. During reconsolidation memories can be strengthened, weakened, or modified, thus opening an opportunity to transform seemingly stable memories [8] and hence treat memory dysfunction across several mental disorders [11–13].

Autobiographical memories frame and shape our emotional life and provide input for planning and facing our everyday challenges. These memories define who we are and generate an updated sense of self [14], thus constituting the milestones of social communication. Autobiographical memory dysfunction is a hallmark of affective disorders and is maybe the main cause for the ruminative retrieval of overgeneral negative information observed in depression [15, 16]. Thus, we hypothesized that the reactivation of autobiographical memories and reconsolidation may lead to the incorporation of new emotional or specific information into the original trace; this mechanism may play a role in psychotherapeutic approaches for MDD [17]. Indeed, under the umbrella of CBT, some innovative psychotherapeutic techniques for the modification of dysfunctional autobiographical memories in depression have been actively investigated [18, 19].

The overarching aims of this review are (1) to provide an overview of autobiographical memory disturbances in depression from a cognitive perspective; (2) to review neuroimaging studies of brain networks disturbed in depression that are also believed to support autobiographical memory processing; and (3) to review emerging evidences of psychotherapeutic techniques targeting autobiographical memory disturbances in depression. We speculate that mechanisms of memory reconsolidation may be explored as a novel target for the modification of dysfunctional autobiographical memories in MDD.

## 2. Search Strategy

For this narrative review, we performed a comprehensive search of Pubmed/MEDLINE and PsycInfo electronic databases from inception to October 10th, 2014. Search terms were “autobiographical memory,” “memory reconsolidation,” “neuroimaging,” “psychotherapy,” “cognitive behavioral therapy” cross-referenced with “depress*∗*.” Only articles published in English were considered. Articles were considered for inclusion based on overall methodological quality. Relevant meta-analyses were also included.

## 3. Autobiographical Memory Disturbances in Depression from a Cognitive Perspective

Several decades of research indicate that individuals with mood disorders remember their past differently from healthy never-depressed controls [20]. The autobiographical memory test (AMT) remains the most widely used instrument for the assessment of autobiographical memory in depression research [21]. In the AMT participants are asked to recollect a specific memory in response to a presented cue word within a predefined time limit (e.g., 30 s or 60 s). The cue words vary in emotional valence and studies often include positive and negative words (e.g., joy and sadness, resp.) [22]. According to their content, specificity, and duration, autobiographical memories are then classified (see the following part).

*Summary of Terms and Definitions Employed in This Review*

*Specific Memories*. These memories refer to autobiographical memories that can be localized in time and space and often do not last longer than 24 hours.

*Extended Memories.* These memories refer to autobiographical memories that extend over long periods of time.

*Categorical Memories. *Autobiographical memories that reflect a repeated event (i.e., cannot be mapped to a specific time and place).

*Semantic Autobiographical Memories.* These refer to memories that form the general knowledge about oneself (i.e., personal semantics).

*Episodic Memories*. These autobiographical memories are characterized by a particular self-reflective mental state, referred to as autonoetic consciousness, which implies that the individual recollects or imagine his/her personal events with a sense of (re/pre) experiencing by mentally “travelling in time,” whether in the past or in the future.

*Strictly Episodic Autobiographical Memories*. These memories are not only spatiotemporally unique autobiographical memories but are also accompanied by subjective (re/pre) experiencing phenomenological details (e.g., sensory, affective, and contextual details).

*Conceptual-Self.* This theoretical mental attribute is stored in the semantic memory system in the form of personal beliefs, values and attitudes, self-knowledge of personality traits, and judgments on a number of categories related to our abstract self-representation.

*Prospection.* Imagining ourselves in the future, or prospection, plays a crucial role in planning, allowing one to select strategic behaviors to engage in successful goal pursuit. Some theorists have argued that remembering and future-oriented thinking may reflect a single mental (brain) process.

*Navigation*. Topographical orientation refers to the capacity to navigate spatial environments imagining one's current position, the desired endpoint, and possible routes using both egocentric and allocentric perspectives.

*Theory of Mind.* A key aspect of social behavior refers to the capability of understanding (i.e., mentalizing) that the behavior of others is motivated by inner states, such as thoughts, emotions, and values. The possession of a theory of mind is necessary to understand our peers (i.e., to take another's perspective to predict their actions and reactions).

*Default-Mode Network (DMN)*. The pattern of brain activations observed during rest conditions had been called the default mode of brain function and may represent stimulus-independent thought or mind-wandering. The DMN may set the stage for self-projection or scene construction.

The disturbed processing of autobiographical memories is a trait-like cognitive manifestation of depression that may contribute to the onset [23–25] and development [26] of the disorder. The next sections discuss the abnormalities of autobiographical memory found in depression.

### 3.1. Biased Recollection of Autobiographical Memories

One striking clinical feature of patients during a major depressive episode is the pervasively negative tone when they refer to their past. In depression, a systematic autobiographical bias favoring negative experiences is a replicated finding [27, 28], with faster retrieval of negative autobiographical memories when cued as well as a heightened spontaneous recollection of negative memories [29, 30]. A selective attention towards negative events may facilitate encoding of negative autobiographical memories [5, 27]. Moreover, a tendency to interpret ambiguous scenarios in a negatively valenced fashion has been reported [31, 32], which may further contribute to the preferential encoding of negative autobiographical memories in depression.

The recall of emotionally positive memories has been identified as a core adaptive emotion mechanism to counteract sad mood [32, 33]. In addition to the biased retrieval of negative memories described above, depression is also accompanied by diminished (and slower) access to positively valenced autobiographical past events [34–36]. Even following recollection of positive autobiographical memories, subjects with a previous diagnosis of depression do not seem to experience mood enhancement [37], and in certain circumstances recall of encouraging personal information may even be detrimental [34, 37]. Moreover, individuals with a past history of depression may recall positive autobiographical memories that are less vivid [38] and less emotionally intense [39] than never-depressed controls. It should be noted that a recent meta-analysis study of AMT data failed to confirm that a significantly biased recall of more negative and fewer positive autobiographical memories occurs in depression when compared with controls [40]. Despite methodological discrepancies [40], this meta-analysis concurs with reports suggesting that recall of overgeneral autobiographic memories in depressive patients in comparison to healthy never-depressed controls (vide infra) is the most consistently replicated finding across studies [29].

### 3.2. Overgeneral Memories

Another evident feature of autobiographical memories in depression is the propensity to recollect categorical memories. In contrast to specific autobiographical episodes, these overgeneral recollections comprise themes related to repeated events, which present a consistent pattern across many past personal experiences. There is now a large evidence base showing that this overgeneral processing pattern overrides the recall of specific time and place details (i.e., episodic recall) [20, 41, 42].

A possible explanation why categorical autobiographical retrieval is so exuberant in depression relies on cognitive theories of depression with their emphasis on the activation of underlying negative schemata in this disorder, which arguably consist of well-consolidated negatively valenced categorical themes [5]. A previous study used a “life chapters” task to investigate the more emotionally salient overgeneral themes in depression [34, 43]. Participants built individual timelines, dividing their autobiographical past into “chapters” (e.g., “time at school,” “time since married,” etc.) and recollected positively and negatively valenced information related to each chapter. Depressive individuals displayed increased coherence and repetition of negative information for each individual chapter. Conversely, never-depressed participants presented the opposite pattern [43]. A greater lifetime number of depressive episodes was related to a lack of positively valenced coherence, indicating that a lack of positive autobiographical themes is a possible marker for episode recurrence [43]. However, these relevant findings need to be confirmed in prospective studies.

There is now compelling evidences that the impairment experienced by depressive individuals to recollect specific autobiographical memories is consistently associated with a worse prognosis (for a meta-analysis see [26]). There is a reciprocal association between the recall of categorical memories in depression and ruminative processes [44]. For example, there are evidences that negatively valenced ruminative content may be instrumental in inducing overgeneral retrieval in depression [16] and in dysphoria [45]. The field awaits the design of longitudinal studies to address causal associations between overgeneral retrieval, rumination, and depression risk. Recent evidences indicate that individuals with elevated scores of* neuroticism* (a personality trait characterized by relatively stable tendencies to respond with negative emotions to threat, frustration, or loss) have the tendency to retrieve negatively biased and overgeneral autobiographical memories [46, 47]. Importantly,* neuroticism* is one of the most consistently replicated personality features to be associated with a higher risk for depression [48, 49]. Thus,* neuroticism* may mediate the relationship between the dysfunctional processing of autobiographical memory and the onset of depression.

### 3.3. Other Psychological Mechanisms Related to Emotional Autobiographical Memories

The recall of emotional autobiographical memories is in certain circumstances a painful process. Explicit and implicit psychological mechanisms to avoid or suppress the assessment of negative past memories and/or the emotions often linked to these memories seem to be more common in depression [50, 51]. However, these mechanisms may be counterproductive, with greater intrusion of unwanted autobiographical memories [52]. Attempts to suppress these unwanted memories may further promote the recollection of other distressing autobiographical memories [52].

Mental avoidance mechanisms may operate in those with depression in the retrieval process of emotional memories. These mechanisms seem to be particularly prominent when those memories are recalled as mental images instead of verbal narratives [53]. Depressive individuals tend to adopt an observer perspective (i.e., they see themselves in the situation but from the perspective of an outsider) when recalling image-based memories [54]. A study in a nonclinical sample demonstrated using contrasting experimental manipulations that imagining positive events from one's own (i.e., field) perspective is critical to improving positive affect [55]. Notwithstanding the fact that this finding deserves replication in a sample with clinical depression, it seems possible that the adoption of an observer perspective as opposed to a field perspective may contribute to depressive mood, regardless of the emotional valence of autobiographical memories.

Efforts to avoid unwanted autobiographical memories and the adoption of an observer perspective may spur ruminative processed focused on the memories themselves or on relating those memories to depressogenic categorical themes through “mental traveling” [56].

### 3.4. The CaR-FA-X Model: An Integrative Model of Autobiographical Memory Processing in Depression

**Figure 1:**
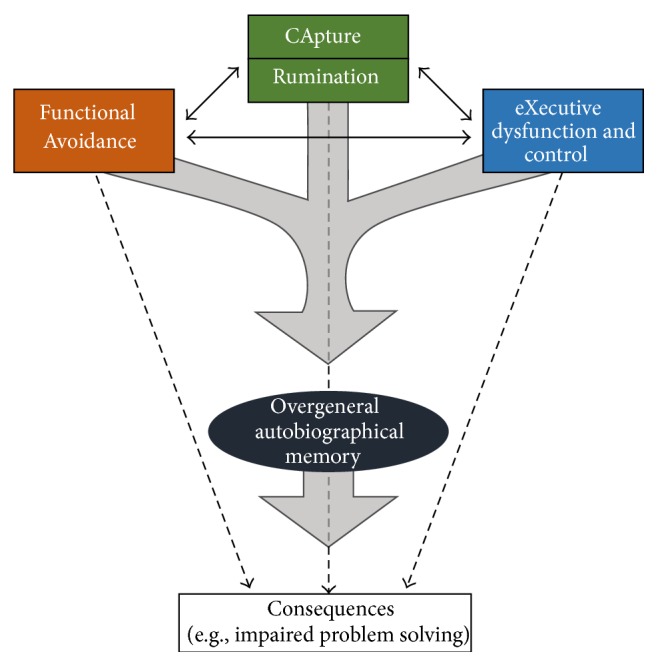
The CaR-FA-X model. Three factors (CApture/Rumination, Functional Avoidance, and impaired eXecutive function and control) interact to decrease the specificity of retrieved autobiographical memories. These less specific memories and the three factors* per se* can then have effects on cognition and behavior.

The CaR-FA-X model (Figure 1) proposed by Williams and colleagues [42] conceptualizes the core mechanisms related to reduced autobiographical memory specificity in depression. This model postulates that difficulties accessing specific autobiographical memories result from the capture (Ca) of memory search efforts by consolidated categorical depressogenic themes, which then engage analytical, evaluative ruminative (R) processes referred to as* brooding* [57]. Such* capture* mechanisms are exacerbated by ingrained functional avoidance (FA) of specific details of distressing autobiographical events, which in turn leads to the processing of an autobiographical representation at the categorical level. The ability to counteract these dysfunctional processing mechanisms is compromised as a function of the limited executive (X) control, which is a consistent feature present in individuals with depression even in remitted states [58, 59].

## 4. Brain Networks Related to Autobiographical Memory Dysfunction in Depression

### 4.1. Brain Networks Involved in Autobiographical Memory Processing in Healthy Subjects

The neurobiological substrates related to autobiographical memory retrieval have been extensively investigated in healthy human individuals through functional neuroimaging studies. Six published meta-analyses have synthesized the main findings related to autobiographical memory retrieval in healthy never-depressed individuals [60–64]. Overall, these studies have shown that autobiographical memory retrieval involves the hippocampus [65–68], lateral temporal cortices [60, 69], anterior cingulate cortex (ACC) [66, 70], and the dorsolateral [69, 71, 72] and ventromedial [73, 74] prefrontal cortices. These findings are summarized in Table 1.

Svoboda and colleagues performed the first of these meta-analyses [60] and found that a core, left-lateralized network of brain regions, including the medial and ventrolateral prefrontal cortex; the medial, lateral, and retrosplenial/posterior cingulate cortices; the temporoparietal junction; and the cerebellum, are primarily involved in AM retrieval. However, this meta-analysis included evidences obtained from different experimental paradigms. The search for mechanisms of autobiographical memory retrieval had followed two distinct theoretical orientations. In the experimental, laboratory-based tradition, subjects might be asked to study a word list and a few minutes later tested on that list. The idea is that each word is a micro-event, and understanding how individuals recall or recognize such micro-events would ultimately inform how life events are recollected. The second tradition is more naturalistic in that researchers investigate real-life past memories. A version of the AMT is often employed in this approach. Therefore, the subsequent analysis performed by McDermott and colleagues aimed to test whether laboratory-based and autobiographical retrieval tasks would differ regarding neurobiological (i.e., in brain areas activated) substrates [61]. Hence this meta-analysis revealed that these two paradigms activate different neural networks while retrieving autobiographical memories (see Table 1 for further details).

Interestingly, Spreng and collaborators [64] synthesized 19 studies and found that brain areas related to autobiographical memory retrieval, prospection, navigation, theory of mind, and the default-mode network (DMN) overlap. Thus, the assessment of autobiographical memory might be probing other mental processes, which are related to self-representation in the past and in the future as well as to theory of mind, although this hypothesis deserves confirmation and replication in future studies. Kim investigated further the role of the DMN and had proposed a dual-subsystem model for the DMN: a cortical midline subsystem (CMS) and a parietotemporal subsystem (PTS) [63]. Areas of the CMS were associated more with an autobiographical memory > laboratory-based memory contrast than with an autobiographical memory > rest contrast, whereas an opposite pattern emerged in PTS regions (i.e., an autobiographical memory > rest contrast was more evident than an autobiographical memory > laboratory-based memory contrast). The author suggested that the CMS subsystem would be more involved in self-reference processing, while the PTS system would be primarily related to memory retrieval* per se*. Nevertheless, this model has some limitations. For example, a reciprocal communication between the CMS and the PTS was not accounted for, while the lack of fine anatomical resolution is a significant shortcoming. However, the model may have heuristic value as it might provide a framework to investigate the role of different brain networks subserving the DMN in the recollection of autobiographical memories.

Martinelli et al. [75] performed three meta-analyses of functional neuroimaging studies investigating neural networks related to the retrieval of* episodic* memories (the authors further studied “strictly” episodic memories),* semantic* memories, and the conceptual-self (Table 1). Importantly, this investigation seems to confirm the prominent role of the ventromedial prefrontal system in self-representation, as this region was consistently related (i.e., activated) in the three domains. Overall, these findings are in accordance with postulations by Conway and Pleydell-Pearce [76] and Conway et al. [77] suggesting that autobiographical memory should be viewed as part of a larger self-memory system with two functions: maintaining adaptive correspondence and ensuring self-coherence.

### 4.2. Brain Networks Involved in Autobiographical Memory Processing in Depression

The aforementioned dysfunctional processing of autobiographical memories in depression and the identification of neural networks related to the recollection of autobiographical memories in healthy human subjects prompted researchers to investigate whether brain activation in depressive patients would differ from the pattern observed in control participants. We identified five relevant functional neuroimaging studies performed in participants with depression compared to healthy controls to date [78–82]. The main findings are depicted in Table 2. Four studies have specifically evaluated brain activation patterns related to autobiographical memories. Zhu and colleagues performed the first study investigating connectivity disturbances in regions involved in the DMN as correlates of autobiographical memory in depression [80]. These authors found that a decrease in functional connectivity between the posterior cingulate cortex and the precuneus (observed in treatment-naïve, first episode depressive individuals) correlated negatively with the retrieval of overgeneral autobiographic memories. Furthermore, in the study by Young and colleagues, differences in the pattern of brain activation associated with the retrieval of specific autobiographical memories were observed in remitted patients with major depressive disorder compared to controls [82], while another study from the same research group found a differential activation of brain structures in first-degree relatives of individuals with MDD [81]. These findings suggest that alterations in brain activation associated with the retrieval of specific autobiographical memories may represent trait markers or even functional neuroimaging endophenotypes for depression.

Overall, all these studies showed that the activation of several brain regions differed when compared to healthy participants, notwithstanding no specific finding consistently emerged across different investigations. Some methodological aspects might have contributed to these inconsistent findings, namely, different clinical characteristics of included participants with MDD (e.g., severity of affective symptoms), previous exposure to antidepressant drugs, as differences in experimental paradigms across studies.

Finally, overgeneral processing of information might be related to two distinct processes: either a decrease in pattern separation or an increase in pattern completion. Pattern separation refers to the capability to dissociate similar stimuli conveyed from the external world in distinct nonoverlapping neuronal representations, while pattern completion enables the proper generalization of similar stimuli conveyed from the external world in the case of a partial sensory input [83, 84]. Converging evidences indicate that the granule cells of the dentate gyrus (DG) of the hippocampus are primarily involved in pattern separation [84], while the CA3 region of the hippocampus has been implicated in pattern completion [85]. Furthermore, extra-hippocampal regions are also involved in pattern separation and in overgeneral memory, including the* nucleus reuniens* and the medial prefrontal cortex [86]. However, the role of all these areas in the encoding or retrieval of autobiographical memories in depression remains to be established.

Taken together, the precise neurobiological substrates subserving autobiographical memory dysfunction in MDD remain unknown (i.e., most findings deserve independent replication, with the proper control of sample characteristics as well as methodological differences). Furthermore, studies investigating brain activation patterns following the retrieval of autobiographical in depression (which may likely reflect reconsolidation mechanisms) are lacking in the literature.

## 5. Manipulations of Autobiographical Memories: Possible Therapeutic Implications for Depression

Moscovitch and Nadel proposed a theory for memory reconsolidation referred to as multiple trace theory (MTT) [87]. According to this theory, the hippocampus remains an integral part of the memory trace and it is always activated during retrieval of episodic memories, regardless of the age of the memory. The MTT suggests that every time a memory is recollected, the underlying mnemonic trace enters a labile state and thus requires another period of consolidation referred to as “reconsolidation” [88]. Such period opens an additional opportunity to transform, update, or even disrupt access to the memory [8]. Notwithstanding memory reconsolidation was far more studied in experimental animals; this phenomenon has also been repeatedly demonstrated in humans, including declarative memories (see [89] for a review).

Schwabe and Wolf [90] attempted to disrupt the reconsolidation of autobiographical memories. On day 1, participants completed an AMT asking them to remember life episodes of the past week. Specifically, they were instructed to associate events to six adjectives (two positive, two neutral, and two negative). One group performed this reactivation of events after they read the story “War of Ghosts” to disrupt the reconsolidation of autobiographical memories. Three other groups performed only the reactivation, only read the story, or did nothing, respectively. A surprise memory recall test one week later showed that the reactivation + interference group remembered significantly less details of the neutral events, but no difference was observed for “positive” or “negative” events. The same authors also demonstrated that exposure to a “socially evaluated cold pressor test” (i.e., to activate a stress response) after the reactivation of autobiographical memories disrupted neutral but not emotionally valenced memories [91]. Perhaps emotional memories would require special conditions for modification because they are stronger and more resistant to change (vide infra).

Lane and colleagues recently proposed an integrative model suggesting that essential changes across diverse psychotherapeutic modalities involve the following: (1) reactivating old (sometimes painful) memories; (2) engaging in new emotional experience that is incorporated to these reactivated memories through reconsolidation; and (3) reinforcing the integrative memory structure by practicing a new way of behaving and experiencing the world in a variety of contexts [17]. This model considers the relevance of emotional arousal in the therapeutic context as well as the intricate and complimentary relationship between* episodic* (autobiographical) memories and the* semantic* memory system [92–94]. Given the relevance of autobiographical memory for implicit/explicit cognitive and emotional processes, research efforts have been directed to develop novel psychotherapeutic strategies specifically targeting autobiographical memory disturbances in depression.

Memory specificity training (MEST) is designed for participants with depression to increase the retrieval of specific past memories, counteracting the recollection of overgeneral autobiographical memories described above. Raes and colleagues [95] developed a group-based MEST program with a sample of depressed inpatients in an uncontrolled trial. The program comprises five sessions conducted by trained psychotherapists, where difficulties in recollecting specific autobiographical memories are exhaustively explored. Through repetitively practicing the recall of specific memories elicited by both positive and neutral cue words in early sessions and to negative cue words in later sessions patients ultimately introduce specific information and then retrieve specific autobiographical memories following the presentation of all types of cues. This pilot trial evidenced that the retrieval style of patients became more specific and improvements in specificity were significantly associated with amelioration of several cognitive processes including rumination, cognitive avoidance, and problem-solving skills [95]. Subsequently, the first randomized controlled trial (RCT) of MEST was conducted in a sample of bereaved, depressed, Afghan refugees living in Iran (*n* = 23); this RCT also included a 2-month followup [19], which at the end evidenced that participants assigned to the MEST group retrieved a higher proportion of specific memories and had lower depression scores. However, this trial had several limitations, including the small sample size and the fact that although included participants had clinically significant depressive symptoms (a score > 27 in the Mood and Feelings Questionnaire was required for participation), a diagnosis of depression was not established with a validated structured interview. Therefore, these encouraging initial findings require replication in a large and well-designed RCT that includes participants with a clearly established diagnosis of depression.

These preliminary yet promising results of the MEST approach may rest on reconsolidation mechanisms, through the updating of overgeneral memories with incorporation of specific information. Thus, we can speculate that its efficacy might be improved with the exploration of some aspects of memory reconsolidation. For example, the total duration of the protocol and/or the cued reactivation of autobiographical memories could be adjusted depending on specific characteristics of the retrieved memory. For instance, it is known that the age and strength of the memory influence whether reactivation induces destabilization followed by reconsolidation [96, 97]. Furthermore, the content and/or subtype of the retrieved autobiographical memory trace might influence the likelihood of modification after reactivation. Rumination could also promote reactivation/reconsolidation cycles, thus opening a “window” for the manipulation of reconsolidation through MEST. Finally, the stress response is able to impair the reconsolidation of autobiographical memories depending on their emotional content [91]. Therefore, controlling physiological parameters of the stress response might be used during MEST sessions to probe any possible interference, while the cold pressor stimulus might be used to enhance specific retrieval to neutral cues.

The impact of recalling positive memories may be enhanced through processes aiming to enrich these memories with affective, visual, and sensory details [34]. For instance, it has been shown that the positive impact of the memories in individuals with depression was enhanced by focusing on detailed aspects of the memories, in contrast to processing them in an abstract way [98]. Accordingly, it has also been shown that when positive autobiographical material is elaborated through imagery, the impact on emotion is potentiated [99, 100]. A psychotherapeutic technique referred to as method-of-loci (MoL) was developed to facilitate assessment of these elaborated autobiographical memories when they are most needed (i.e., in the service of emotion regulation on a day-by-day basis).

**Figure 2:**
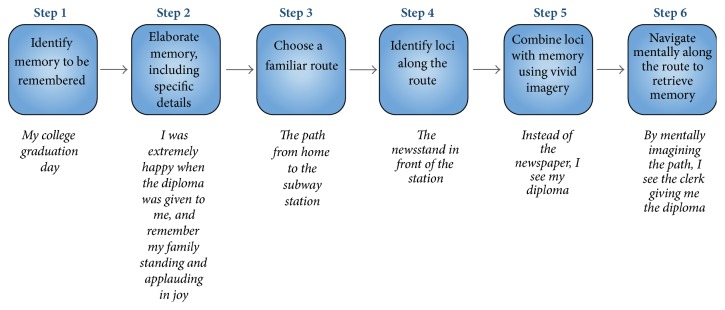
The method-of-loci (MoL). Associating a memory to loci in a familiar route might be used to enhance the retrieval of positive autobiographical memories in depression.

The method-of-loci (MoL) is an ancient mnemonic method that relies on memorized spatial relationships between loci that are used to arrange and recollect episodic memories [34]. The basic paradigm aims to incorporate visual imagery to each to-be-recollected piece of information with one of the loci along a route. MoL significantly ameliorated memory performance in naïve participants [101, 102]. In an initial, nonrandomized study, MoL was compared to a chunking-and-rehearsal technique in small sample of participants with major depressive disorder [18]. Participants completed a week of retrieved training until the point they could recollect all their identified memories without error. On a surprise recall test after a further week, only participants allocated to MoL training exhibited a “ceiling” memory recollection. Notwithstanding, the MoL is a promising and simple tool to enhance the assessment of elaborated positive memories in depression; these findings deserve replication in a larger randomized trial.  Figure 2 depicts a hypothetical worked example of this approach. The MoL involves the incorporation of new information into an existing memory trace, which may in turn involve reconsolidation mechanisms. Thus, a careful scrutiny of the conditions under which the reactivation and updating is conducted may (at least in theory) improve its overall efficacy (vide supra).

Mindfulness-based cognitive therapy (MBCT) may be an effective therapeutic modality for depression [103]. Although MBCT is not primarily targeted at memories* per se*, MBCT aims to enhance affective executive control over mental life (including autobiographical memories) through the practice of meditation skills that promote the ability to “step back” from painful (i.e., distressing) mental content [104]. The psychological changes promoted by MBCT are supported by emerging neurobiological evidences [105].

The practice of MBCT requires a highly trained psychotherapist [104]. Thus, research efforts have been directed to distill core cognitive elements of MBCT into simpler protocols. Kross and colleagues [106] investigated the effects of self-distancing, the process of intentionally stepping back on an experience to reflect on it and reappraise it from the perspective of a distant observer. This more reflective process differs from simply adopting and observer perspective upon autobiographical memories, which could be counterproductive, as discussed above. Preliminary evidences indicate that analyzing the meaning of memories from a self-distanced perspective may promote psychological benefits for people with depression [106].

Previous studies showed that blood oxygen-level-dependent (BOLD) activity in the amygdala increased in response to both positive and negative emotional stimuli in healthy individuals [107]. A functional lateralization between the right and the left amygdala has been proposed such that the right is activated in rapid/automatic detection of emotional stimuli, while the left enables detailed stimulus evaluation [107, 108]. Evidences now suggest that hemodynamic responses in the left amygdala may be “doubly dissociated” in depression from healthy controls by virtue of presenting a greater response to negative stimuli and an attenuated response to positive stimuli [109, 110]. Recently, Young et al. [111] developed a novel real-time functional magnetic resonance imaging neurofeedback (rtfMRI-nf) training of amygdala activity in patients with MDD. Participants were assigned to receive rtfMRI-nf training from either the left amygdala (*n* = 14) or the horizontal segment of the intraparietal sulcus (control group, *n* = 7) and instructed to contemplate positive autobiographical memories to raise the level of a bar representing the hemodynamic signal of the brain region of interest to a target level. Participants in the experimental group upregulated their amygdala responses during memory recollection [111]. Significant pre-post scan improvements in positive mood were evidenced in the experimental group versus the control group. These promising preliminary data deserve independent replication in a larger sample, and the long-lasting effects of left amygdala rtfMRI-nf training on mood remain to be established. Furthermore, these findings suggest the usefulness of this technique to manipulate amygdala responses during the reconsolidation of autobiographical memories.

## 6. Concluding Remarks and Perspectives

This review indicates that autobiographical memory dysfunction (especially, overgeneral memory recollection) is a constant neuropsychological correlate of depression. Furthermore, compelling evidence indicates that these disturbances may represent trait-markers for the disorder. Discrete brain regions integrating separate networks mediate the retrieval of autobiographical memories. These networks are distinctly activated during the recollection of autobiographical memories in depression, although a consistent pattern of activation in comparison with healthy individuals did not emerge. Finally, this extensive review indicates that promising therapeutic strategies specifically targeting autobiographical memory dysfunction in depression have been developed. However, these techniques are based on a solid preliminary research base, and more well-designed trials are needed to establish the effectiveness of these interventions before incorporating them in the routine care of depressive patients. We hypothesize here that the retrieval of autobiographical memories in depression would render the memory trace labile and susceptible to change through the process of reconsolidation. Furthermore, ongoing research on biobehavioral mechanisms of memory reconsolidation in humans may provide valuable insights to apprimorate psychotherapeutic strategies targeting autobiographical memory disturbances in MDD.

Our review also opens important directions for further research. For example, additional studies are needed to elucidate brain networks subserving autobiographical memory dysfunction in depression. Despite drug therapies targeting the reconsolidation of autobiographical memories being abundant in posttraumatic stress disorder (PTSD) (see [10] for a review), these studies are still lacking in depression. To date, no published drug trial had attempted to modulate the reconsolidation of distressing autobiographical memories in depression. Furthermore, the role of subsyndromal affective symptoms on the persistence of autobiographical memory disturbances in depression deserves elucidation. Future studies should include larger samples controlling for potential confounders (e.g., treatment status, number of previous episodes, etc.).

Finally, disturbances in autobiographical memory processing seem to cut traditional diagnostic boundaries and are present in several chronic mental disorders (e.g., substance abuse, PTSD, and depression). The recently proposed National Institute of Mental Health research domain criteria (RDoC) [112] state that targeting transdiagnostic, neurobiologically informed domains could improve precision and guide therapeutic efforts in psychiatry in the future. In this changing* scenario*, disturbed autobiographical memory neural circuits could represent a novel transdiagnostic therapeutic target for mental disorders.

## Figures and Tables

**Table 1:** Meta-analyses of functional neuroimaging studies which investigated brain networks involved in autobiographical memory (AM) processing in healthy subjects.

Reference	Number of included studies (*N*)^∗^	Meta-analysis method	Details of the studies/participants	Main findings
Svoboda et al., 2006 [60]	24 studies (*N* = 243)	“Effect location”	Detailed characteristics of the studies participants (e.g., age, gender) not reported Inclusion criteria (1) scanning occurred at the stage of memory retrieval (2) retrieval involved the recollection of episodic AMs that were personally experienced, relatively remote, and specific in time and in place; (3) included at least one contrast in which a reference task was compared with the AM condition	AM recollection activated a left-lateralized network, which included the mPFC, lPFC, TPJ, and retrosplenial/posterior cingulate cortex. The cerebellum (predominantly the right) was also activated

McDermott et al., 2009 [61]	18 studies (*N* not reported)	ALE	Detailed characteristics of studies participants not reported Inclusion criteria (1) included a voxelwise (i.e., whole-brain) contrast for data of interest (2) reported areas of peak activation in a standardized coordinate space (3) neurologically normal (4) young adults (i.e., no patient populations or older adult participants)	Laboratory-based and autobiographical memory retrieval tasks active largely nonoverlapping brain networks. For example, laboratory-based studies display left-lateralized activations within frontal and parietal cortices (in areas not activated by AM retrieval); both tasks activated regions within the PCC

Spreng et al., 2009 [64]	19 studies (*N* = 228)	ALE	Detailed characteristics of studies participants not reported	This meta-analysis revealed a significant overlap between brain areas involved in AM recollection, prospection, navigation, theory of mind, and the default-mode network (DMN); less than a quarter of investigated clusters were domain-specific; the mPFC and lateral temporal regions were activated in the five domains

Kim, 2012 [63]	37 studies (*N* = 494)	ALE	Detailed characteristics of studies participants not reported Inclusion criteria (1) healthy participants (2) performed a whole-brain analysis (3) reported coordinate-based analyses of the data (4) performed at least one of the four contrast types relevant the analysis	This meta-analysis proposed a functional subdivision for the DMN namely a “cortical midline subsystem” (CMS) represented by the anteromedial prefrontal cortex and the PCC and a “parietotemporal subsystem” (PTS); a double dissociation model was proposed in which the CMS plays a critical role to self-processing, whereas the PTS is more related to memory retrieval *per se *

Viard et al., 2012 [62]	58 studies (*N* = 866)	ALE	Age range: 15–77 years Inclusion criteria (1) performed voxelwise contrasts (2) used univariate or multivariate analysis approaches with uniform significance and cluster size thresholds applied throughout the brain (3) reported standard-space stereotactic coordinates	This meta-analysis demonstrated that (1) specific cues tend to activate more the right anterior hippocampus compared to the use of generic cues; (2) recall/imagine tasks activated more the left posterior parahippocampal gyrus compared to recognition tasks; (3) (re/pre) experiencing strictly episodic events tends to activate more the bilateral posterior hippocampus compared to episodic events; (4) older individuals displayed a greater activation of the right anterior hippocampus compared to younger ones, and (5) “strictly” episodic events triggered by specific cues elicited greater left posterior hippocampal activation compared to episodic events triggered by specific cues

Martinelli et al., 2013 [75]	38 studies (*N* = 575)	ALE	Inclusion criteria (1) measured regional cerebral blood flow or oxygenation, or glucose metabolism (2) include whole-brain statistics (3) reported coordinates in a standard reference frame (4) healthy subjects (5) young adults (mean range: 18–59 years) (6) used auditory and visual cues for retrieval (7) included independent of the emotional valence	Three separate meta-analyses were performed; areas activated by episodic AMs were the hippocampus and bilateral parahippocampal formation, the precuneus, the PCC, and left middle temporal gyrus; areas activated by semantic AMs were the ACC, PCC, left superior and middle temporal gyrus, left thalamus, left fusiform gyrus, and parahippocampus; the “conceptual self” activated the ACC. The three domains (i.e., episodic AMs, semantic AMs, and conceptual self) activated the mPFC suggesting that this structure is crucial to self-representation

ACC = anterior cingulate cortex; AM = autobiographic memory; ALE = activated likelihood estimation; mPFC = medial prefrontal cortex; lPFC = lateral prefrontal cortex; TPJ = temporoparietal junction; PCC = posterior cingulate cortex. ^∗^Some individual studies were included in more than one meta-analysis.

**Table 2:** Functional neuroimaging studies which investigated brain networks involved in autobiographical memory (AM) processing in individuals with depression.

Reference	Sample size (*N*)	Sample characteristics^∗∗^	Neuroimaging approach/task	Main findings
Whalley et al., 2012 [78]	15 individuals with MDD 15 controls	Age and education matched Age: controls > MDD Gender ratio: controls = MDD	1.5 T fMRI/recognition task	Participants with MDD displayed a lower activation of the right middle frontal cortex and bilateral inferior frontal gyrus, with only the right inferior frontal gyrus meeting the stricter cluster extent threshold

Young et al., 2012 [79]	12 unmedicated individuals with MDD 14 controls	MDD: 4 females, age = 34 ± 11, WASI = 120 ± 15 Controls: 7 females, age = 29 ± 9, WASI = 118 ± 12	3.0 T fMRI/computerized AM test	Activation of the left hippocampus/striatum and right parahippocampal gyrus was higher for AM recall than a subtraction task in HC but lower in MDD; activation of the anterior insula bilaterally was lower for specific AM recall versus subtraction with the magnitude of the decrement being higher in MDD

Zhu et al., 2012 [80]	35 individuals with a first MDE^∗^ 35 matched controls	MDE: 18 females, age = 20 ± 2 Controls: 19 females, age = 20 ± 2	1.5 T fMRI/AMT	Participants with depression exhibited increased functional connectivity between the medial prefrontal cortex and ACC and decreased functional connectivity in the PCC/precuneus; the increased functional connectivity in the PCC/precuneus correlated negatively with OGM

Young et al., 2013 [81]	16 healthy controls (HC) 16 individuals at-risk for MDD (HR) 16 individuals with MDD (MDD)	HC: 11 females, age 36 ± 10, WASI = 114 ± 10 HR: 11 females, age 33 ± 11, WASI = 109 ± 7 MDD: 11 females, age 38 ± 10, WASI = 115 ± 9	3.0 T fMRI/computerized AM test	During recollection of specific AMs compared to example generation, the following differences were noted: (1) Right Medial Frontal Polar Cortex: MDD > HR and MDD > HC (2) Right Frontal Operculum: HC > HR and MDD > HR (3) Right Pregenual ACC: MDD > HC and MDD > HR (4) Left Pregenual ACC: MDD > HC and MDD > HR (5) Left Cuneus: HR > MDD; HR > HC; MDD > HC and HR > MDD

Young et al., 2014 [82]	16 healthy controls (HC) 16 formerly depressed individuals (rMDD) 16 individuals with current depression (cMDD)	HC: 10 females, age = 27 ± 8, WASI = 111 ± 10 rMDD: 10 females, age = 32 ± 12, WASI = 110 ± 9 cMDD: 10 females, age = 34 ± 9, WASI = 104 ± 9	3.0 T fMRI/computerized AM test	During recollection of specific AMs compared to example generation, the following differences were noted: (1) Right lateral OFC: rMDD > HC and rMDD > cMDD (2) Right inferior temporal gyrus: rMDD > HC and rMDD > cMDD (3) Right parahippocampus/hippocampus: rMDD > HC and rMDD > cMDD (4) Left DMPFC: cMDD > rMDD; cMDD > HC and rMDD > HC (5) Left parahippocampus/hippocampus: cMDD > rMDD; cMDD > HC and rMDD > HC (6) Left anterior insula: cMDD > rMDD; cMDD > HC and rMDD > HC

MDD = major depressive disorder; MDE = major depressive episode; fMRI = functional magnetic resonance imaging; HC = healthy controls; ACC = anterior cingulate cortex; PCC = posterior cingulate cortex; OGM = overgeneral autobiographical memories; HR = individuals at risk for MDD; OFC = orbitofrontal cortex; rMDD = remitted MDD; cMDD = current major depressive episode; WASI = Wechsler Abbreviated Scale of Intelligence. ^∗^Treatment-naïve; ^∗∗^female count in the sample; age: mean ± SD (years); WASI: mean ± SD.
